# Public health round-up

**DOI:** 10.2471/BLT.21.010121

**Published:** 2021-01-01

**Eliminating cervical cancer**

Primary schoolgirls in Masaka, Rwanda comfort each other while receiving their human papillomavirus vaccination, joining a growing population of young people who can hope for a future free of cervical cancer. On 17 November****WHO launched a global strategy to accelerate the elimination of cervical cancer following the adoption of a resolution at the Seventy-third World Health Assembly in 2020.

**Figure:**
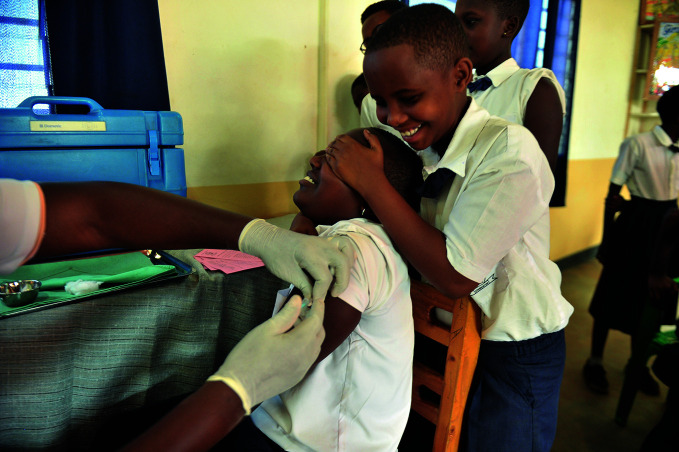
UNICEF/Laurent Rusanganwa

## COVID-19 humanitarian response

The United Nations (UN) and partners released an overview of plans designed to respond to the humanitarian crisis resulting from the COVID-19 pandemic. The UN estimates that 235 million people worldwide will need humanitarian assistance and protection in 2021 – an increase of 40% on people requiring such assistance in 2020. The UN-coordinated plans aim to reach 160 million of the people most in need of life-saving support and will cost an estimated US$ 35 billion.

Presented on 1 December, the *Global humanitarian overview 2021* sets out 34 response plans covering 56 vulnerable countries. UN Secretary-General António Guterres called on the world to “stand with people in their darkest hour of need”.

https://bit.ly/3a8Xz9U

## Targeting cervical cancer

The World Health Organization (WHO) launched a global strategy to accelerate the elimination of cervical cancer. Released on 17 November, the strategy outlines three key steps relating to vaccination, screening and treatment.

The launch of the strategy represents a historic milestone, it being the first time that 194 countries have committed to eliminating a cancer, following the adoption of a resolution at the Seventy-third World Health Assembly in 2020.

“Eliminating any cancer would have once seemed an impossible dream, but we now have the cost-effective, evidence-based tools to make that dream a reality,” said WHO Director-General Tedros Adhanom Ghebreyesus. He stressed however that realizing the benefits of the tools available will require unrelenting determination to scale up their use.

https://bit.ly/3gEBJfG

## Leading causes of death and disability

Noncommunicable diseases constituted seven of the world’s top 10 causes of death in 2019. This is according to WHO’s *Global health estimates* which were published on 9 December.

Covering the period 2000 to 2019, the estimates reveal trends in mortality and morbidity caused by diseases and injuries.

Heart disease remains the number one cause of death, accounting for almost 9 million deaths in 2019, up from around 7 million deaths in 2000.

Among other trends identified are a sharp increase in dementia-related deaths (including deaths related to Alzheimer’s disease) rising from 584 000 to 1.6 million, and diabetes-related deaths, which increased from 878 000 to 1.5 million globally between 2000 and 2019, with an 80% increase in deaths among males.

The estimates highlight the need for an intensified global focus on preventing and treating cardiovascular diseases, cancer, diabetes and chronic respiratory diseases, as well as tackling injuries, in all regions of the world, as set out in the agenda for the UN sustainable development goals.

https://bit.ly/3qQeww6

## Africa unready for COVID-19 immunization

The countries of the WHO African Region are largely unprepared for the roll-out of COVID-19 vaccines in 2021. This is according to new WHO research based on a Vaccine Readiness Assessment Tool that was sent to all 47 countries in the WHO African Region in 2020.

Intended for use by ministries of health, with support from WHO and the United Nations Children’s Fund, the assessment tool covers 10 key areas, ranging from planning and coordination to community engagement. Some 40 countries responded, generating an average score of 33% readiness for a COVID-19 vaccine roll-out, well below the desired benchmark of 80%. “African governments must urgently ramp up readiness,” said Dr Matshidiso Moeti, WHO Regional Director for Africa, adding that planning and preparation are key to achieving success.

https://bit.ly/342I67s

## Ebola outbreak ends

The Minister of Health of the Democratic Republic of the Congo declared the end of the most recent Ebola virus disease outbreak. The declaration was made on 18 November in accordance with WHO recommendations, 42 days after the last confirmed case tested negative for the second time on 6 October 2020 in Makanza Health Zone, Equateur Province.

WHO congratulated the dedicated responders who tracked cases, provided treatment and vaccinated more than 40 000 people at high risk of exposure. Key to the response’s success was the use of innovative cold chain storage that kept the Ebola vaccine at temperatures as low as minus 80 °C and enabled responders to vaccinate people living in communities without electricity.

https://bit.ly/37OvBh6

## First emergency use listing for a vaccine

WHO listed the novel oral polio vaccine type 2 (nOPV2) for emergency use to address increasing cases of a vaccine-derived polio strain in a number of African and Eastern Mediterranean countries. The 13 November listing was the first time an emergency use listing was applied to a vaccine.

WHO developed the emergency use listing process to expedite the assessment and approval of unlicensed medical products needed in public health emergencies. The procedure was introduced during the West Africa Ebola virus disease outbreak of 2014–2016, when multiple Ebola diagnostics received emergency use listing; since then, numerous COVID-19 diagnostics have also been listed in this way. It is expected that the procedure will play a key role in making COVID-19 vaccines accessible quickly.

https://bit.ly/3m4JWLz

## Malaria progress stalls

There were an estimated 229 million malaria cases in 2019, a number that has remained virtually unchanged over the past four years. According to WHO’s *World malaria report 2020*, published on 26 November, progress on malaria mortality also stalled, the disease claiming some 409 000 lives in 2019 compared with 411 000 in 2018.

As in previous years, the African Region accounted for more than 90% of the overall disease burden, despite reducing malaria-related mortality by 44% since 2000, from an estimated 680 000 malaria deaths per annum to 384 000 in 2019.

Lack of funding for malaria is a major concern. In 2019, for example, total funding reached US$ 3 billion against a global target of US$ 5.6 billion.

https://bit.ly/37T8YYW

## Putting a value on health

WHO announced the formation of a new Council on the Economics of Health for All. Staffed by leading economists and health experts, the council will aim to support a proactive Health for All economic agenda, by framing the commitment of resources to national, regional and global health systems as investments in the future rather than as short-term costs.

The council will be chaired by economist Mariana Mazzucato, Professor in the Economics of Innovation and Public Value and Founding Director of the Institute for Innovation and Public Purpose at University College London.

“The COVID-19 pandemic has demonstrated the consequences of chronic under-investment in public health. But we don’t just need more investment; we must also rethink how we value health,” said WHO Director-General Tedros Adhanom Ghebreyesus, announcing the creation of the council on 13 November.

Mazzucato stressed the need for a proactive agenda, putting well-being and inclusion at the centre of how we create, distribute and measure value. “We are living through multiple crises: economic, climate and health related,” said Mazzucato. “If we continue to patch up the system each time, we will always be one step behind.”

https://bit.ly/342uidb

**Cover photo**

An elementary school student washes her hands before lunch in Darkhan-Uul Province, Mongolia.

**Figure:**
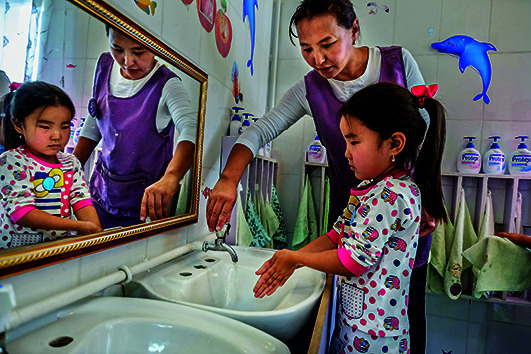
WHO/Yoshi Shimizu

## Tackling the infodemic

WHO called on key stakeholders and the global community to commit to addressing the challenges posed by the infodemic, aspects of which are undermining the COVID-19 response.

Setting out a seven-point action plan, WHO called on stakeholders and the global community to support a whole-of-society approach and engage with communities in the production, verification and dissemination of information that leads to healthy behaviours during epidemics and pandemics.

https://bit.ly/3nlHbXm

## WHO Foundation appoints CEO

The WHO Foundation appointed Anil Soni as its inaugural Chief Executive Officer, effective 1 January 2021. Soni is tasked with leading the Foundation in its efforts to invest in innovative, evidence-based initiatives that support WHO efforts to ensure healthy lives and promote well-being for all.

The WHO Foundation is an independent grant-making agency with headquarters in Geneva which was launched in May 2020 to act as a platform for new types of public–private engagement, while protecting WHO’s neutrality and independence as the world’s leading international health authority.

https://bit.ly/3gBtCAJ

**Looking ahead**

28 January–3 February. The Prince Mahidol Award Conference. Theme: COVID-19, Advancing towards an equitable and healthy world. https://bit.ly/37T50j5

1–9 February. WHO Executive Board meeting. WHO headquarters, Geneva, Switzerland. https://bit.ly/37SZ9Kt

22–25 February. The Global Vaccine and Immunization Research Forum. https://bit.ly/3m8jh0c

